# Climatic Treatment of Consumption

**Published:** 1883-07

**Authors:** 


### CLIMATIC TREATMENT OF CONSUMPTION.

THE New York Medical Journal of June 16th gives the opinions of a number of eminent medical men, in different sections of the country, in regard to the effects of climate in pulmonary consumption.

Dr. Austin Flint, of New York, says: It does not appear from the analyses of my cases (670 cases of consumption) that changes of climate have, in a marked degree, a beneficial influence as compared with the hygienic measures available at home. Hence it seems a fair inference that the benefit derived from the change is due, not so much to a climatic influence, *per se*, as to the circumstances incidental to the change.

Dr. A. L. Loomis, New York, says, in his work "Diseases of the Respiratory Organs:" "Experience shows that one individual improves in a warm, moist air, another improves in a cold, dry air. When a dry, cold atmosphere is indicated, direct your patient to make a trial of such a climate as Minnesota, but let none go there who are unable to exercise in the open air. The climate of Southern California, Colorado, certain parts of Georgia, and South Carolina is well adapted to that class of patients who require a *dry warm atmosphere.* Persons in the incipient stages do well in Colorado, but the air is so thin and dry it may cause hemorrhage, if they have previously existed where a *cool and moderately moist climate* is required. Dr. Loomis highly recommends the Adirondack region.

*The climatic treatment of consumption is almost exclusively confined to its first stages.* In this stage patients will sometimes be benefited by a long sea voyage.

Dr. Samuel G. Armah, of Brooklyn, says: In cases of primary tuberculosis, in which the constitutional symptoms of mal nutrition outrun the local lung lesions, I prefer a dry, stimulating tonic atmosphere, such as is found in Minnesota, Dakota, Montana, New Mexico, or the eastern slope of the Rocky Mountains. I am not partial to Florida.

Dr. H. D. Didama, Syracuse, N. Y., says: These are briefly my rules:

"1. If possible, make a change early.

"2. Go where the temperature is satisfactory to the patient—invigorating or soothing, as the case may be.

"3. In incipient cases with bronchial catarrh in apex, a mountain elevation and proper instructions will, by securing chest expansion, do good.

"4. Home for the hopeless."

Dr. William T. Plant, Syracuse: "I incline to the sthenic climates for *incipient phthisis.* I think *very* few *actual* consumptives recover in Florida or the West Indies. Many recover in Colorado, Minnesota, etc. My opinion on this point has been formed from considerable observations, extending over eighteen years. I have directed patients to the elevated and dry regions of the far West rather than south to the Atlantic States. The result has almost always been gratifying when the patient has gone early. I have *known* several consumptives to go *early* to Florida, Nassau, etc. and they have never done well. Some have come back after a few months and gone West, where they improved. I think it quite likely that *some* cases are benefited by leaving our rigorous climate and making of Florida a *winter* residence."

Dr. Louis Elsberg, New York: "A sedative climate for patients with sensitive mucous membranes; a sthenic climate for catarrhal tendencies. In summer to Adirondack Mountains, Catskills, or at least where there are trees, horseback exercise and animal food. In winter, southern equable climates, where the same requisites of beef, air and exercise can be supplied."

Dr. J. N. Danforth, Chicago: "I prefer a cool climate with some constant incentive to exhilarating exercise. With my phthisical patients any change is tentative, and they so understand it; but a sthenic climate seems to benefit the greatest number. Travel, change, novelty and the consequent excitement, generally benefit, and I think, form the main parts in climato-therapy."

Dr. James P. Ross, Chicago: "Favor a stimulating, tonic climate—Southern Californa or San Antonio, Tex. I send patients away in winter and spring."

Dr. Roswell Park, Chicago: "Prefer sthenic climates. I tell patients to go where the conditions of a *temperate* climate, with proper *dryness* and freedon from sudden changes can be found, no matter if the locality has no 'reputation.' I like the mountain region of Tennessee."

Dr. Alonzo B. Palmer, Ann Arbor, referred to his recent work on "Practice of Medicine," in which he says: "There must be a certain good degree of digestive power, or a prospect of soon obtaining it. . . . Comparative youth and vigor are required. In comparatively early periods of chronic cases there can be no doubt of the beneficial effects of elevated, dry, cool, electrical, stimulating, climatic situations (Rocky Mountains, Col.). For those more impressible and delicate, New Mexico. In bronchial, pneumonia and other inflammatory cases, a sojourn in Florida, Santa Barbara or Georgia. In the treatment of ordinary cases of chronic tuberculosis, *cool* and *dry* climates give better results than *warm* and *moist*. A particularly equable temperature must not be insisted upon except in bronchial and inflammatory cases, as equability presupposes moisture, which is far less favorable to the tuberculous condition."

Dr. Jay Owens, St. Paul, Minn.: "Do not favor a sedative climate. Florida is covered with consumptives' graves. A sthenic climate, such as Minnesota in summer, and the Valley of the Rio Grande River, in New Mexico, in winter. Dryness is the great curative factor. Patients do not take cold in a dry atmosphere, if they are reasonably careful. From personal experience, I know a

*dry* atmosphere to be of great advantage. I know of many cases of recovery by those afflicted coming to Minnesota early in the disease. I send my patients to Socorro, N. M., in the winter, and they come back much improved."

Dr. D. W. Hand, St. Paul: "Favor a sthenic climate. Direct patients to Las Vegas, N. M.; to Aiken, S. C.; Thomasville, Ga."

Dr. George F. French, Minneapolis, Minn.: "Sedative climate for hæmorrhagic cases. Have saved a wife, pronounced incurable by several eminent diagnosticians, by changing climate from Portland, Maine, to Minneapolis. Here three years."

Dr. James T. Whittaker, Cincinnati: "Experience teaches me to prefer Nassau to all southern climates; because it is a pure sea air is the reason for the good effects, I believe. For other cases—the higher and colder the better, if general comfort can be secured—Colorado, about Denver. If I had the disease myself, I should, in the light of existing knowledge, go and *live* in the coldest, driest climate I could find, as in Switzerland near the line of perpetual snow."

We have thus given the views of a number of physicians on the question of climate for consumptives, and might have quoted several more from the same article. It will be observed that there is no material difference of opinion among them as to the course to be advised under the different conditions of the disease. They all favor a change of climate; to a cold, dry climate in the early stage of the disease; and in the advanced stage to a southern climate. Now we fully recognize the advantages of a change of location, not only in cases of lung disease, but in nearly all diseases where a change would be practicable, for hygienic reasons if for no other; but it does not seem to us essential that a patient should be sent a long distance from home and friends, to procure the exercise and fresh air necessary to the healing process. We believe that the majority of patients affected with lung disease would do better with proper care and treatment at home. We believe that in its first stage almost every case is curable, by means calculated to counteract the unfavorable symptoms as they present themselves, while the system is being strengthened by proper food and regular and vigorous exercise in the open air.

---

A STORY is told of a woman in the rural districts who wanted to keep up appearances, and who was often thwarted in this by her innocent and matter-of-fact daughter. One day, when a visitor was present at the table, the hostess said to her daughter, "Where are all our knives?" "Here they are, both of them," was the astounding reply.

